# An Anatomical Study Using Computed Tomography, Magnetic Resonance Imaging, and Rhinoscopy of the Nasal Cavity of Domestic Cat (*Felis silvestris catus* L.) and Big Cats: Lion (*Panthera leo leo* L.), Leopard (*Panthera pardus kotiya* L.), and Cheetah (*Acinonyx jubatus jubatus* S.)

**DOI:** 10.3390/ani14081172

**Published:** 2024-04-13

**Authors:** Elena Díaz Martínez, Alberto Arencibia Espinosa, Marta Soler Laguía, David Kilroy, Francisco Martínez Gomariz, Diego Luis Casas García, Cayetano Sánchez Collado, Francisco Gil Cano, José Raduán Jaber, Gregorio Ramírez Zarzosa

**Affiliations:** 1 Department of Anatomy and Comparative Pathological Anatomy, Veterinary Faculty, University of Murcia, 30100 Murcia, Spain; elena.diaz2@um.es (E.D.M.); f.gomariz@colvet.es (F.M.G.); scollado@um.es (C.S.C.); cano@um.es (F.G.C.); 2 Department of Morphology, Anatomy and Embriology, Veterinary Faculty, University of Las Palmas de Gran Canaria, Transmontaña, Arucas, 35416 Las Palmas, Spain; alberto.arencibia@ulpgc.es (A.A.E.); joseraduan.jaber@ulpgc.es (J.R.J.); 3 Department of Medicine and Surgery, Veterinary Faculty, University of Murcia, 30100 Murcia, Spain; mtasoler@um.es; 4 UCD Veterinary Science Centre, University College Dublin, D04 V1W8 Dublin, Ireland; david.kilroy@ucd.ie; 5 Centro Veterinario de Mínima Invasión Canarias CVMIC, 35019 Las Palmas de Gran Canaria, Spain; dlcasas@gmail.com

**Keywords:** CT, MRI, endoscopy, rhinoscopy, nasal cavity, anatomy, feline, big cats

## Abstract

**Simple Summary:**

The nasal cavity has been studied extensively in dogs. However, in cats, there are few studies of the specific anatomy of the nasal cavity, and as far as big cats are concerned, we have not currently found any studies. This work describes the anatomy of the nasal cavity of the domestic cat and three big cats (leopard, lion, and cheetah) using computed tomography, magnetic resonance, and rhinoscopy. The aim of this study is to highlight the specific anatomical characteristics of each of these species, as well as the differences between the domestic cat and its nondomestic congeners. This study highlights the advantages and disadvantages that each imaging technique provides for the study of the nasal cavity. This will help veterinary clinicians diagnose the pathologies that can affect this anatomical region and thereby improve their treatment. In addition, these big cats are in a vulnerable state, close to extinction, and better diagnosis and treatment of their pathologies would improve the survival of these species.

**Abstract:**

The objective of this work was to study the normal anatomy of the nasal cavity of the three species of big cats (leopard, lion, and cheetah) compared to the domestic cat through the use of computed tomography, magnetic resonance imaging, and rhinoscopy. Computed tomography allowed us to clearly visualize the entire bony and cartilaginous framework that supports the nasal cavity. Magnetic resonance imaging permitted better visualization of the soft tissues of this cavity. On the other hand, rhinoscopy enabled the direct visualization of the mucosa of the vestibule and nasal cavity, which is very useful in the diagnosis of masses or foreign bodies. Furthermore, with this technique, it has been possible to observe several small orifices from the nasolacrimal duct, the pharyngeal auditory tube, and the lateral nasal gland. Computed tomography, magnetic resonance imaging, and rhinoscopy are useful tools in analysis of the anatomical characteristics of the nasal cavity in these species.

## 1. Introduction

The nasal cavity is the first region of the respiratory tract. It begins with the nostrils, openings for air entry, and ends with the nasal choanae [1]. The nasal conchae are several delicate bony structures that are referred to as conchae in the rostral nasal cavity and turbinates in the caudal nasal cavity [2]. These conchae create spaces between them, the nasal meatuses (dorsal, middle, ventral, and common), through which inhaled and exhaled air flows [3]. Caudoventrally, the nasal cavity communicates with the nasopharynx through the choanae or nasopharyngeal openings [4]. The nasal cavity is divided into two halves by the nasal septum. Caudally, this septum is osseous and fuses with the cribriform plate of the ethmoid bone, and it becomes cartilaginous as it extends rostrally. In addition, the ventral part of the nasal septum is supported by the vomer bone. The cartilaginous nasal septum cannot be identified in radiographs, but it can be distinguished in computed tomography (CT) and magnetic resonance imaging (MRI). Moreover, it should be noted that cats have frontal sinuses, lateral maxillary recesses, and small sphenoidal sinuses [5].

The nostrils of carnivores are developed in a nasal plane, represented by the vertex of the nose. The vertex of the nose is usually moist; however, it lacks glands [6]. Most of the respiratory tract is covered with ciliated pseudostratified epithelium and a varying number of goblet cells containing mainly serous glands. In the caudal part of the nasal cavity (the olfactory region), the mucosa is specialized for olfaction and contains the olfactory nerve endings and glands. The submucosa of the respiratory region has several vascular plexuses. This vascularization warms the air and, by causing evaporation of the glandular secretions, adds moisture to the inhaled air [4]. Therefore, this complex architecture composed of conchae, turbinates, and meatuses serves to increase the surface area of the nasal passages, thus enhancing the main functions of olfaction, filtration, humidification, and warming of the nasal cavity [2].

The nasal cavity is the area that is most frequently affected by diseases of the upper respiratory tract. A complete knowledge of this region is of great importance, since pathologies in the nasal cavity of cats are common, among which neoplasms, infectious and non-infectious inflammatory diseases, trauma, dental diseases, and nasopharyngeal diseases predominate [2,7,8,9].

Since its inception, radiology has been the most widely used technique for the study of the nasal cavity. However, this technique has several drawbacks that limit and make the study of the nasal cavity difficult, and the main one is the overlapping of structures [9,10]. Nevertheless, CT and MRI are techniques that allow for a more reliable study of the nasal cavity and, therefore, its associated pathologies. Both techniques are more sensitive than radiology and more accurate in determining the extent of disease [9]. De Rycke et al. [11] reported that all the structures of the nasal cavity can be identified by both CT and MRI studies. The major advantages that CT can provide over MRI are the reduction in both the time and the cost to carry out the tomographic study [11]. In fact, CT is considered the best imaging technique for an initial evaluation of diseases of the nasal cavity [1]. In contrast to CT studies, MRI allows for the differentiation between soft tissues and fluids and the generation of multiple images without having to reposition the patient [5,9,11]. However, the use of CT or MRI in certain pathologies, such as neoplasms or chronic nasal diseases, does not allow for a clear definitive diagnosis, and it is necessary to take a tissue biopsy (performed by endoscopy) [12]. In this context, the rhinoscopy of the nasal cavity is particularly important. This imaging technique displays the internal structures of the nasal cavity, detects anatomical abnormalities such as fungal plaques and neoplasms, makes it possible to take biopsies, and aids the localization and extraction of foreign bodies [3,13]. Nevertheless, prior knowledge of the normal anatomy of the nasal cavity accelerates and simplifies an endoscopic study [14].

In addition, the leopard, the lion, and the cheetah are species that are threatened with extinction according to the Red List of Threatened Species of the International Union for Conservation of Nature (IUCN). Due to the declining population of these big cats, all three species are classified as vulnerable [15,16,17]. For this reason, it is important to conduct these anatomical studies and consider the respective pathologies of these species of large cats.

In the veterinary literature, there are few reports on the use of CT, MRI, and rhinoscopy in the study of the anatomy of the feline nasal cavity, since most of the published studies are based on the dog as the species of interest [1,2,3,14,18]. Only Conchou et al., Losonsky et al., and Gil Cano et al. [9,10,19] studied the specific anatomy of the feline nasal cavity using CT and MRI. However, to the best of the authors’ knowledge, there are no publications that use rhinoscopy to study the anatomy of the feline nasal cavity. Most of the relevant published articles only studied certain specific clinical cases using CT, MRI, or rhinoscopy [13,20,21,22,23,24,25,26,27,28,29].

To date, there is no anatomical atlas of the morphology of the nasal cavity of big cats. Only a few studies have been published regarding the anatomy of the tiger head, brain, tarsal, and stifle joints using MRI [30,31,32]. There are just a few published studies on certain clinical cases that affect this region in large cats (nondomestic cats) [33,34,35,36]. In this context, our study provides a comprehensive anatomical atlas of the nasal cavity of the domestic cat, as well as of the leopard, lion, and cheetah, using CT, MRI, and rhinoscopy and highlights the interspecific felid structural differences. Advanced knowledge of the anatomy of the nasal cavity of these species through imaging techniques will provide veterinary clinicians with better diagnostic skills, as well as a better understanding of the respiratory physiology of each species, by knowing their anatomical differences.

## 2. Materials and Methods

### 2.1. Animals

A total of six felids were used in this study. Three big cat specimens, a lion (*Phantera leo leo*), a leopard (*Phantera pardus kotiya*), and a cheetah (*Acinonyx jubatus jubatus*), were obtained from the Terra Natura’s natural park (Murcia, Spain). They were euthanized for reasons beyond our study. Before performing the necropsies, the common carotid arteries and external jugular veins were cannulated. After the necropsies, all feline heads were sectioned at the level of the C3-C4 intervertebral space. The heads were then fixed in an embalming solution (10% formaldehyde (1.5 L), glycerine (3 L), methyl alcohol (12.5 L), and phenol (1 L)) for 7 days under refrigeration. Finally, the three heads of the big cats were used for CT, MRI, and rhinoscopic studies. In addition, a crossbreed male cat cadaver (*Felis silvestris catus*), 3.8 kg and approximately 3.5 years old, euthanized for reasons not related to this study, was obtained from the Zoonoses Service of Murcia (Murcia, Spain). The cat head was used to obtain a sagittal section for the anatomical study (level sections) and to compare the anatomical structures of this region with the heads of the big cats. In this cat, the same pre-necropsy procedure was carried out as described above, but subsequently, the vasculature was injected with colored epoxy to better visualize the arterial and venous supply. Blue epoxy was used for the venous system and was injected via the external jugular veins, and red epoxy was used for the arterial system and was injected via the common carotid arteries (E20 plus BIODUR^®^, Biodur Products, Biodur, Heidelberg, Germany).

The CT and MRI study of the domestic cat were carried out on a pet cat with the prior consent of its owners (they were informed about the study and signed the consent form). It was a healthy 2-year-old male cat, weighing 3.5 kg. The rhinoscopic study was carried out on another cat, again after obtaining the informed consent of its owners (they were also informed about the procedure and signed the consent form). In this case, it was a 4-year-old male cat, 4 kg in weight, and without any health problems. For the CT and MRI study of the cat, the anesthetic protocol consisted of premedication with methadone (Semfortan^®^ 10 mg/mL, Dechra, Northwich, UK) (0.1 mg/kg IV) and induction with propofol (Propofol-Lipuro 1%, Braun, Kronberg, Germany) (4–6 mg/kg IV), followed by volatile isoflurane (Forane, Abott, Chicago, IL, USA) (2–3%) in a flow of 0.8 L/min of O_2_, and mechanical ventilation was used. For the rhinoscopic study, the following anesthetic protocol was used: premedication with medetomidine (Sedator^®^ 1 mg/mL, Dechra) (0.025 mg/kg IM), methadone (Semfortan^®^ 10 mg/mL, Dechra) (0.4 mg/kg IM), and midazolam (Dormazolam^®^ 5 mg/mL) (0.1 mg/kg IM); induction with propofol (Propofol-Lipuro 1%, Braun) (dose response IV), followed by volatile isoflurane (Forane, Abbott) (2%).

This study was supervised, and the research protocol was authorized by the Animal Ethical Committee of Veterinary Medicine of the University of Murcia, Spain (REGA ES300305440012 CEEA: 305/2017; extended on 25 July 2022 as project Type II).

### 2.2. Computed Tomography

CT was performed using a 16-row scanner (Toshiba Astelion, Toshiba Medical System, Madrid, Spain). Big cats and feline heads were placed in a ventral recumbency, with the mandibles parallel to the table using positional aids on the scanner. We used the following technical parameters for all four species: kVp 120, acquisition matrix 512 × 512, spiral pitch factor 0.625, and slice thickness 1.25 mm. A different mA was used depending on the species, ranging from 35 to 150 mAs.

The transverse original data were stored and transferred to the CT workstation. All images were analyzed using the DICOM viewer software (RadiAnt DICOM Viewer 2023.1; Medixant Co., Poznan, Poland). Images were assessed using a bone algorithm (window width, WW > 1500 UH; window level, WL: 400–500 UH).

### 2.3. Magnetic Resonance Imaging

MRI was acquired with a high-field (1.5 Tesla) scanner (General Electric Sigma Excite, Schenectady, NA, USA), and a human head coil with 8 channels was used. For the timing of data acquisition, the heads of the big cats and the domestic cat were placed in ventral recumbency, same as in the CT examinations.

For the MRI study of the feline nasal cavity, we used the MRI parameters shown in Table S1 (Supplementary Materials).

### 2.4. Rhinoscopy

A fixed rhinoscopy unit (Karl Storz^®^, Tuttlingen, Germany) with a camera processor (Karl Storz^®^ image 1 hub, camera head Karl Storz^®^ Image 1 H3 HD, a Storz power led 175) was used to obtain the rhinoscopic images of the nasal cavity of the big cats. A forward telescope (0° enlarged view, diameter 3 mm, length 14 cm, with incorporated fiber optic light transmission) or a forward-oblique telescope (30° enlarged view, diameter 2.7 mm, length 18 cm, with incorporated fiber optic light transmission) was employed for the nose depending on the width of the specimen’s nasal cavity.

To carry out rhinoscopy on the domestic cat, a fixed rhinoscopy unit (Karl Storz^®^, Tuttlingen, Germany) with an endoscopy tower with an HD monitor (9619 NB—Karl Storz^®^), a camera unit (Telecam One-Chip 20212023—Karl Storz^®^), a video unit (Telecam SL II 202130 20—Karl Storz^®^), a 100 W xenon cold light source (XENON 100 SCB 201326 20—Karl Storz^®^), and a forward telescope (diameter 1.9 mm, 3 Fr working channels, 30° enlarged view) was used.

The rhinoscopic procedures were performed on the feline specimens following standard protocols. Each specimen was placed in ventral recumbency and facing the endoscopist. Initial image acquisition was of the external nose morphology, using an optic of 3 mm diameter and 0° vision angle. The rhinoscopic study of the four species was carried out by introducing the rhinoscope into the left nasal orifice. Following this, the nasal cavity was examined by introducing an optic of 2.7 mm and a 30° vision angle, protected by a 3 mm sheath, visualizing first the vestibule, then turning the optic to obtain a complete exposition of this part of the nasal cavity, observing the alar folds. After visualizing the nasal vestibule, the rhinoscope was introduced directly towards the common nasal meatus, which allowed us to observe structures such as the nasal conchae (cornetts), the ethmoturbinates, the maxillary recess, and the lateral nasal gland.

### 2.5. Anatomic Evaluation

The interpretation of the CT, MRI, and rhinoscopic study of the cats was carried out after an exhaustive consultation of various atlases of head anatomy [4,6,19,37,38,39,40]. We tried to accurately describe the anatomical structures of the nasal cavity, while always following the veterinary anatomical nomenclature.

## 3. Results

### 3.1. Computed Tomography and Magnetic Resonance Imaging

Eleven CT, as well as T1-weighted and T2-weighted MRI, figures of each of the feline species were selected based on their relevance to identify important anatomical nasal structures. The similarity of the section in the nasal cavity was prioritized for a greater resemblance between the CT and the MRI study. This is due to slight differences, which may appear within the same level section. The attenuation and signal intensity levels of the structures depending on whether a CT or MRI study was performed are identified in Table S2 (Supplementary Materials).

**Figure 1:**
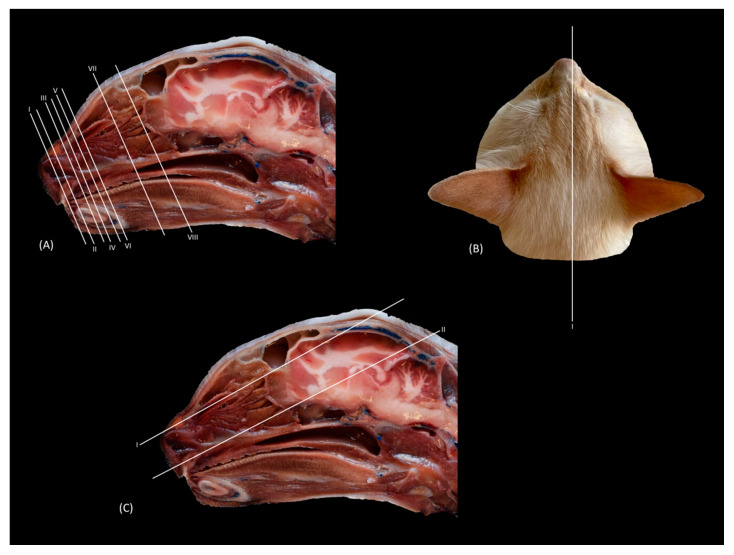
Approximate anatomical level sections of the cat head. Lines depict the transverse (**A**), parasagittal (**B**), and dorsal (**C**) planes. Each number represents the location for each CT and MR transverse (I to VIII), sagittal (I), and dorsal (I–II) images.

**Figure 2:**
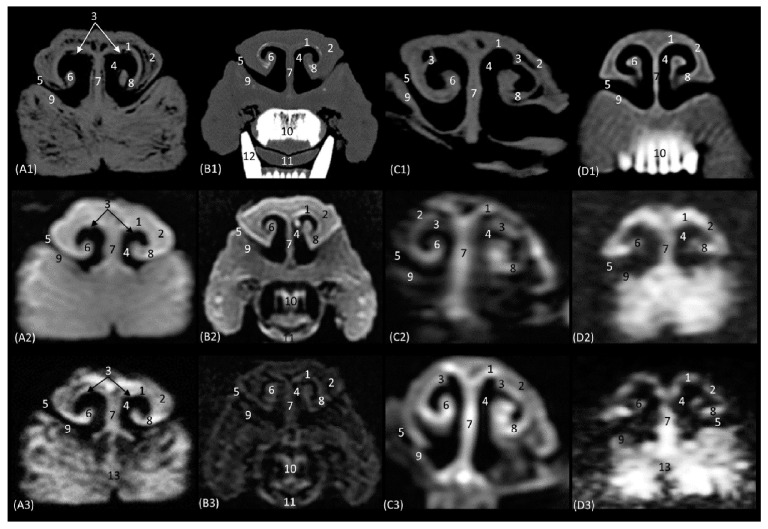
Representative transverse CT (**A1**–**D1**), T1-weighted MR (**A2**–**D2**), and T2-weighted MR (**A3**–**D3**) images at the level of the nasal orifices (nasal plane). Level section (**A**) I. Images are oriented so that the left side of the head is to the right and the dorsal is at the top. All views are rostral. (**A1**–**A3**): Leopard; (**B1**–**B3**): lion; (**C1**–**C3**): cheetah; and (**D1**–**D3**): cat. 1. Dorsal lateral nasal cartilage; 2. nasal wing; 3. parallel folds; 4. nasal orifice; 5. alar groove; 6. alar fold; 7. nasal septum: cartilage; 8. ventral lateral nasal cartilage; 9. lateral accessory nasal cartilage; 10. incisive bone and upper incisive teeth; 11. tongue; 12. lower canine tooth; 13. subnasal groove.

The anatomical level sections of the cat heads are shown in Figure 1. At the level of the nasal plane (Figure 2(A1–D3)), the development of the entire cartilaginous structure that forms the framework of the nose could be identified in the four studied feline species: dorsal and ventral lateral cartilage, lateral accessory cartilage, alar fold, and its corresponding alar groove, with an intermediate degree of attenuation and signal intensity in CT and MRI, respectively. In this context, it is worth highlighting the greater difficulty in identifying nasal cartilages on MRI compared to CT. On MRI, they are visualized as hyperintense lines with respect to the nasal wings that make up the opening of the nose. In contrast, it was observed that the conformation of the alar fold is more elongated and vertical in the lion and the cat, who have a much narrower space in the opening of the nose, than in the leopard and cheetah. Parallel folds were only identified in the leopard and the cheetah. The subnasal groove was visualized in the leopard by means of CT and MRI (Figure 2(A1–A3)) and less clearly in the cat by means of MRI (Figure 2(D3)).

**Figure 3:**
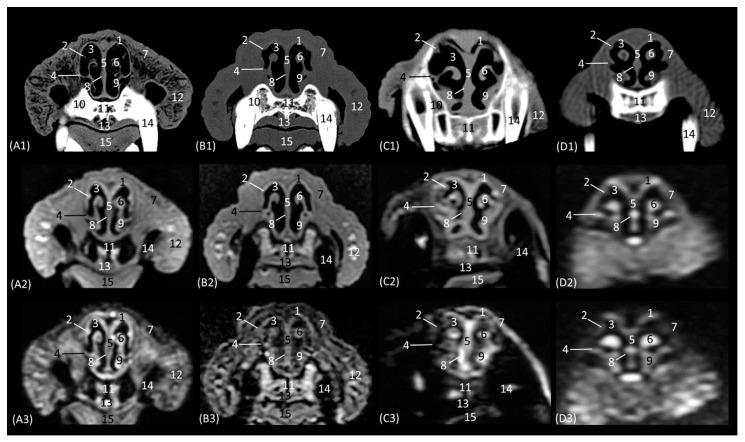
Representative transverse CT (**A1**–**D1**), T1-weighted MR (**A2**–**D2**), and T2-weighted MR (**A3**–**D3**) images at the level of the nasal vestibule. Level section (**A**) II. Transverse images are oriented so that the left side of the head is to the right, and the dorsal is at the top. All views are rostral. (**A1**–**A3**): Leopard; (**B1**–**B3**): lion; (**C1**–**C3**): cheetah; and (**D1**–**D3**): cat. 1. Dorsal lateral nasal cartilage; 2. straight fold; 3. nasal vestibule; 4. lateral accessory nasal cartilage; 5. nasal septum: cartilage; 6. alar fold; 7. nasal wing; 8. nasal cavernous plexuses; 9. basal fold; 10. maxillary bone; 11. incisive bone; 12. upper lip; 13. hard palate; 14. root of the upper canine tooth; 15. tongue.

The next plane shows the nasal vestibule (Figure 3(A1–D3)). The ventral limit of the beginning of the nasal cavity is established at the level of the incisive bone in the four species, with the cortical and medullary cavity being well differentiated on CT. The root of the upper canine tooth is also identified, as well as the maxillary bone. Unlike the CT study, MRI permitted the observation of the incisive bone with a hypointense cortical and a hyperintense trabeculated medullary cavity due to the fat content of the bone marrow. This view shows how, in contrast to the other three species, the maxillary bone of the cheetah already begins to project dorsally as the scan sequence progresses. The incisive bone forms the hard palate at the level of the nasal vestibule. At this section level, we can more easily observe the structures making up the nasal cartilages of the four species, observing hyperintense lines on MRI with respect to the surrounding tissue. The straight fold in the four species at this level is still poorly developed. However, the alar fold is very evident in this section, being much more curved in the cheetah than in the three other felids, in which it develops more vertically. The basal fold is much more developed in the domestic cat than in the other species. On the other hand, in the domestic cat, it can be observed that, proportionally, the nasal wing and the alar fold have a greater depth, since at the same level of the nasal vestibule, these two structures can be identified only in domestic cats (Figure 3(D2,D3)). The nasal cavernous plexuses already begin to become evident in the middle of the nasal septum, being less noticeable at this level in the lion than in the rest of the species studied.

**Figure 4:**
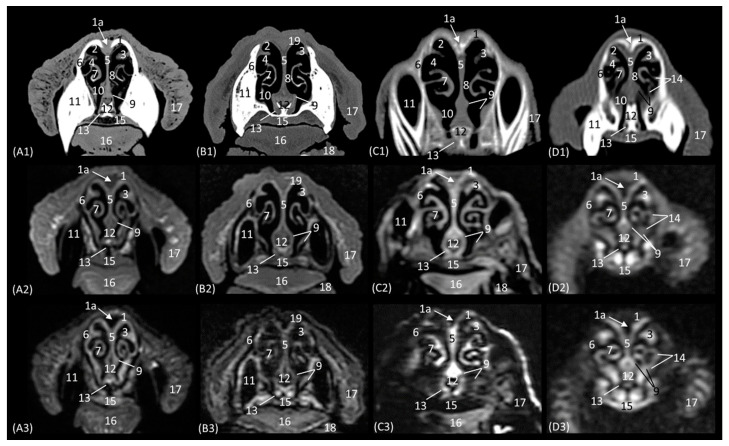
Representative transverse CT (**A1**–**D1**), T1-weighted MR (**A2**–**D2**), and T2-weighted MR (**A3**–**D3**) images at the level of the rostral portion of the respiratory part. Level section (**A**) III. Transverse images are oriented so that the left side of the head is to the right and the dorsal is at the top. All views are rostral. (**A1**–**A3**): Leopard; (**B1**–**B3**): lion; (**C1**–**C3**): cheetah; and (**D1**–**D3**): cat. 1. Nasal bone; 1a. nasal bone: nasal symphysis (arrow); 2. dorsal nasal meatus; 3. dorsal nasal concha: straight fold; 4. middle nasal meatus; 5. nasal septum: cartilage; 6. maxillary bone; 7. ventral nasal concha; 8. common nasal meatus; 9. nasal cavernous plexuses; 10. ventral nasal meatus; 11. root of the upper canine tooth; 12. vomer bone; 13. vomeronasal organ; 14. conchal crest of the maxilla; 15. hard palate; 16. tongue; 17. upper lip; 18. lower lip; 19. dorsal lateral nasal cartilage.

The next section delves into the respiratory portion of the nasal cavity, since the nasal conchae are already beginning to be observed with intermediate attenuation and signal intensity in CT and MRI, respectively (Figure 4(A1–D3)). The bones that make up the floor, side walls, and roof of the nasal cavity are the incisive and vomer, maxillary, and nasal bones, respectively. At this section level, the maxillary bone can already be seen vertically, limiting the nasal cavity laterally and with the nasal bone marking its dorsal border. In the leopard and the lion, the most dorsal part is not visible, since the closure of the nasal bones extends more caudally in these two species. On MRI, both this dorsal limit and the junction between the maxillary bone and the nasal bone are difficult to differentiate, since both are hypointense. Within the nasal cavity, we can see the turbinates or conchae, the vomeronasal organ, and the nasal cavernous plexuses. The dorsal nasal concha of the big cats is identified as flat with only minimal extension into the nasal cavity. In the domestic cat, its development is proportionally much greater. The ventral nasal concha, with a dorsal and a ventral coil, is much more developed than the dorsal nasal concha, and it is slightly ossified at this level in all four species. This concha presents a similar morphology among the species of big cats, having a single start from the conchal crest of the maxillary bone while, at this level, a double start was identified in the domestic cat. These conchae create spaces in the nasal cavity: the dorsal, middle, ventral, and common meatuses, which are observed to be hypoattenuated in CT and hypointense in MRI. In the MRI study, they are not fully labelled to allow for better visualization of the rest of the structures. These spaces are described in all four species, but it is in the lion that they reach the greatest amplitude. At this level, the vomeronasal organ is very well identified; a hypoattenuated duct can be seen in the four species that we studied on CT, while on MRI, it appears slightly hypointense on T1-weighted and barely distinguishable on T2-weighted images. The vomer bone is located medially and has a groove for the support of the cartilage of the nasal septum. On CT, it is very well visualized in a hyperattenuated manner; however, on MRI, a small external hypointense cortical line is displayed, and internally, a hyperintense area, with much greater signal intensity, is seen in the T2-weighted images. It is attached to the maxillary bone and is clearly identified on CT, but on T1 and T2-weighted MRI, this bone is hypointense and is viewed with difficulty. The morphology of this bone is a “Y” shape in the leopard and the lion and more vertical in cats, with a double Y shape and being centrally fused with a stool shape in cheetah. The nasal cavernous plexuses at this section level are well distinguished dorsally and laterally to the vomer bone and are less developed on the lateral walls of the nasal cavity.

**Figure 5:**
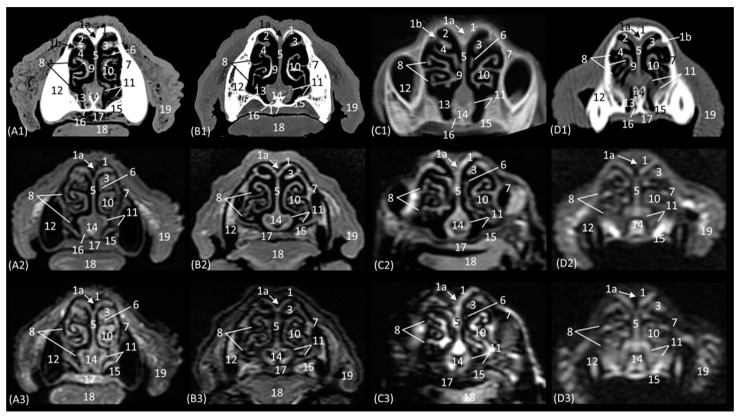
Representative transverse CT (**A1**–**D1**), T1-weighted MR (**A2**–**D2**), and T2-weighted MR (**A3**–**D3**) images at the level of the dorsal and ventral nasal conchae (rostral part). Level section (**A**) IV. Transverse images are oriented so that the left side of the head is to the right and the dorsal is at the top. All views are rostral. (**A1**–**A3**): Leopard; (**B1**–**B3**): lion; (**C1**–**C3**): cheetah; and (**D1**–**D3**): cat. 1. Nasal bone; 1a. nasal bone: nasal symphysis (arrow); 1b. nasal bone: ethmoidal crest (arrow); 2. dorsal nasal meatus; 3. dorsal nasal concha; 4. middle nasal meatus; 5. nasal septum: cartilage; 6. 3rd endoturbinate; 7. maxillary bone: body; 8. maxillary recess; 9. common nasal meatus; 10. ventral nasal concha; 11. nasal cavernous plexuses; 12. root of the upper canine tooth; 13. ventral nasal meatus; 14. vomer bone; 15. maxillary bone: palatine process; 16. vomeronasal organ; 17. hard palate; 18. tongue; 19. upper lip.

In the next section of the nasal cavity (Figure 5(A1–D3)), the conchae begin to appear slightly hyperattenuated on CT. The bones that make up the limits of the nasal cavity at this level are the palatine processes of the maxilla and the vomer bone at the base, the body of the maxillary bone laterally and dorsally, together with the nasal bone. It can be observed that the nasal bone is already closed dorsally in the leopard, while in the lion, it is not yet completely closed at this level. Within the nasal cavity, we can identify the dorsal nasal concha, the ventral nasal concha, and the third endoturbinate, as well as the nasal cavernous plexuses and the vomeronasal organ. At this level in CT, it is observed that the dorsal nasal concha is hyperattenuated in the leopard and the cheetah and slightly in the cat, but in the lion, the tissue continues to show an intermediate attenuation. With MRI, it is more difficult to identify this ossification, but a more hyperintense signal can be observed in the more cranial sections. This bony attachment of the dorsal nasal concha to the lateral wall of the nasal cavity is represented by the ethmoidal crest of the nasal bone. At this level, in addition, the maxillary recess begins to be observable in the four species. The nasal cavernous plexuses continue to be identified in all four species, which is also evident in the ventrolateral wall, with less development in the lion and the cheetah. The third endoturbinate also begins to appear at this section level between the dorsal and the ventral nasal concha in the leopard, the cheetah, and the cat, but not yet in the lion. The vomeronasal organ in the leopard is a large-caliber duct, unlike in the cheetah and the domestic cat. It is topographically next to the long central stem of the vomer bone, which is concave and supports the base of the cartilage of the nasal septum, while in the lion, the concavity of the groove is formed without a bone stem from the maxillary bone.

**Figure 6:**
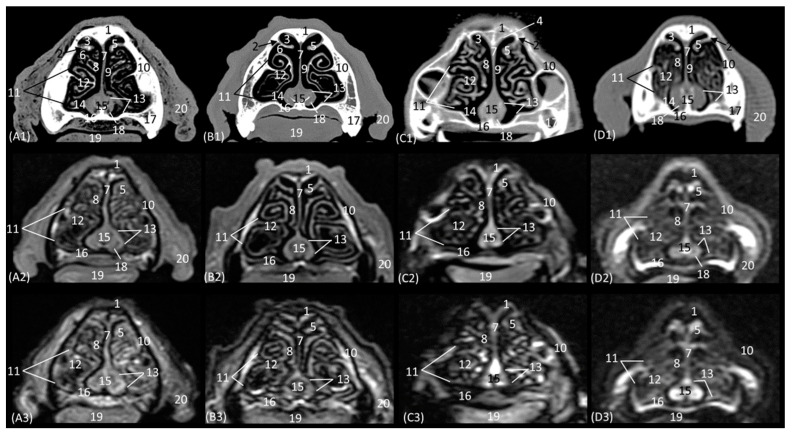
Representative transverse CT (**A1**–**D1**), T1-weighted MR (**A2**–**D2**), and T2-weighted MR (**A3**–**D3**) images at the level of the dorsal and ventral nasal conchae (middle part) and maxillary recess. Level section (**A**) V. Images are oriented so that the left side of the head is to the right and the dorsal is at the top. All views are rostral. (**A1**–**A3**): Leopard; (**B1**–**B3**): lion; (**C1**–**C3**): cheetah; and (**D1**–**D3**): cat. 1. Nasal bone; 2. nasal bone: ethmoidal crest (arrow); 3. dorsal nasal meatus; 4. ethmoid bone: tectorial plate; 5. dorsal nasal concha; 6. middle nasal meatus; 7. nasal septum: cartilage; 8. 3rd endoturbinate; 9. common nasal meatus; 10. maxillary bone: body; 11. maxillary recess; 12. ventral nasal concha; 13. nasal cavernous plexuses; 14. ventral nasal meatus; 15. vomer bone; 16. maxillary bone: palatine process; 17. infraorbital canal; 18. vomeronasal organ; 19. tongue; 20. upper lip.

The next level is represented in Figure 6(A1–D3). Figure 6(C1) shows the section where the external plate of the frontal bone ventrally relates to the ethmoid bone and frontal sinus in the cheetah, since at this level in this species, the nasal bone is no longer sectioned. In the nasal cavity, it is worth highlighting the presence of the infraorbital canal. It can be identified both on CT and MRI in a rounded area that is hypoattenuated and hypointense, respectively, with respect to the maxillary bone. In this section, the third endorturbinate is observed, entering and intertwining between the dorsal and ventral nasal concha. It is evident again in these images that the nasal conchae and endoturbinates in the lion are much thinner than in the other three species, resulting in much wider meatuses. In this section, we can, again, see how the ventral nasal concha in the domestic cat has two bony starts from the maxilla, unlike the big cat species, which only have one.

**Figure 7:**
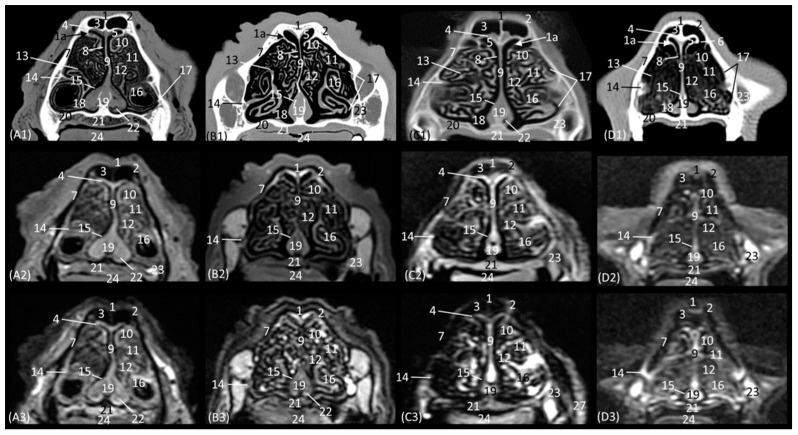
Representative transverse CT (**A1**–**D1**), T1-weighted MR (**A2**–**D2**), and T2-weighted MR (**A3**–**D3**) images at the level of the dorsal and ventral nasal conchae (caudal part). Level section (**A**) VI. Transverse images are oriented so that the left side of the head is to the right and the dorsal is at the top. All views are rostral. (**A1**–**A3**): Leopard; (**B1**–**B3**): lion; (**C1**–**C3**): cheetah; and (**D1**–**D3**): cat. 1. Nasal bone; 2. frontal bone: squama (external plate); 3. frontal sinus; 4. ethmoid bone: tectorial plate; 5. dorsal nasal meatus; 6. frontal sinus: opening; 7. maxillary bone: body; 8. common nasal meatus; 9. nasal septum: cartilage; 10. dorsal nasal concha; 11. middle nasal concha; 12. 3rd endoturbinate; 13. middle nasal meatus; 14. conchal crest of maxillary bone; 15. nasal cavernous plexuses; 16. ventral nasal concha; 17. maxillary recess; 18. ventral nasal meatus; 19. vomer bone; 20. maxillary bone: palatine process; 21. hard palate; 22. vomeronasal organ; 23. infraorbital canal; 24. tongue.

A more caudal section of the nasal cavity is depicted in Figure 7(A1–D3). The bony framework that supports the nasal cavity at this level is composed dorsally of the nasal bone (only in the lion, as it extends more caudally), the external lamina of the frontal bone (squama), the maxillary bone (palatine process), and the vomer. In addition, the zygomatic bone is observed in the lion and in the domestic cat. At this level, the tectorial plate of the ethmoid bone can already be seen in the leopard and cheetah, although it is not yet visible in the lion, delimiting the frontal sinus ventrally, which also occurs in the domestic cat in a more rostral view (Figure 6(D2,D3)). In the lion, the frontal sinus develops much further caudally than in the other species. In addition, only in felids does the frontal sinus present a lateroventral opening towards the nasal cavity. Moreover, the bones of the roof (nasal and frontal bones) have a much greater thickness in lions than in the other species. The vomer bone in this plane already appears in its full extension: in large cats, it is composed of two long vertical arms and a short trunk, while in the domestic cat, it develops with a much longer trunk and shorter arms. In the nasal cavity, we observed the dorsal, middle, and ventral nasal conchae; the third endoturbinate; the vomeronasal organ; the cavernous plexuses; and the infraorbital canal. The vomeronasal organ was observed with a wide duct in the cheetah and a small duct in the leopard, and a duct was not observed in the lion or the cat. In this cross-section, the middle nasal concha is identified for the first time but is camouflaged by the extensive development of the third endoturbinate at this level. The ventral nasal concha shows ventral coiling and is dorsally flattened in the leopard. In the lion, the dorsal and ventral conchae are large but narrow, and in cheetahs and cats, they are smaller.

**Figure 8:**
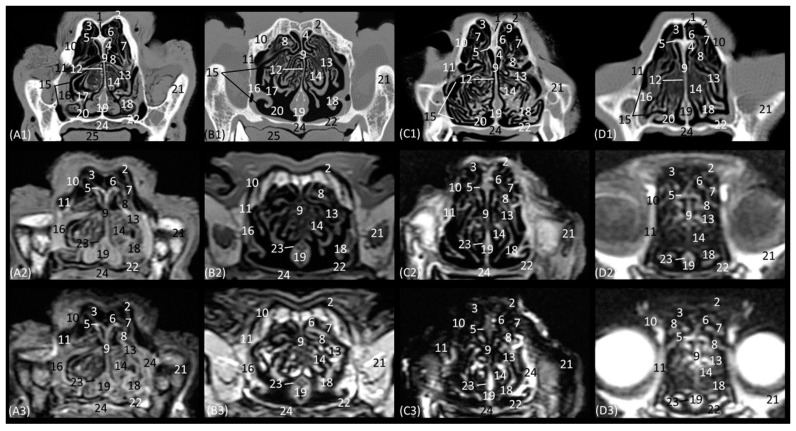
Representative transverse CT (**A1**–**D1**), T1-weighted MR (**A2**–**D2**), and T2-weighted MR (**A3**–**D3**) images at the level of the ethmoid labyrinth and lateral nasal gland. Level section (**A**) VII. Transverse images are oriented so that the left side of the head is to the right and the dorsal is at the top. All views are rostral. (**A1**–**A3**): Leopard; (**B1**–**B3**): lion; (**C1**–**C3**): cheetah; and (**D1**–**D3**): cat. 1. Frontal sinus: septum; 2. frontal bone: squama (external plate); 3. frontal sinus; 4. dorsal nasal meatus; 5. ethmoid bone: tectorial plate; 6. 2nd ectoturbinate; 7. 3rd ectoturbinate; 8. dorsal nasal concha; 9. nasal septum: cartilage/ethmoid bone (perpendicular plate); 10. frontal bone: orbital surface; 11. lacrimal bone: orbital surface; 12. common nasal meatus; 13. middle nasal concha; 14. 3rd endoturbinate; 15. maxillary recess; 16. lateral nasal gland; 17. middle nasal meatus; 18. ventral nasal concha; 19. vomer bone; 20. ventral nasal meatus; 21. zygomatic bone; 22. palatine bone; 23. nasal cavernous plexuses; 24. hard palate; 25. tongue.

The next section level is shown in Figure 8(A1–D3), which allow the zygomatic bone to be identified in all species. The bony structures that make up the nasal cavity at this level is established dorsolaterally by the external and orbital surface of the frontal bone, the lacrimal bone, and the zygomatic bone; ventrally by the vomer bone, as well as the palatine bone; ventrally to the frontal sinus by the tectorial plate of the ethmoid bone; and medially by the cartilage of the nasal septum. This section also highlights the significant thickness of the frontal bone in lions, which is unlike the other feline species. It is observed to be hyperattenuated on CT, while on MRI, it is observed with a hypointense cortical with a hyperintense medullary cavity. Furthermore, at this level, the frontal sinus does not yet appear in the lion, while in the rest of the species, the bony septum of the frontal sinus can already be seen, and inside the nasal cavity, the first, second, and third ectoturbinates are already identifiable. The nasal septum is already ossified at this level, since it appears to be hyperattenuated on CT and hyperintense on MRI, although only very slightly in the lion. The bony component of the hard palate at this section is provided by the palatine bone. Within the nasal cavity at this level, we highlight the three ectoturbinates, the dorsal, middle, and ventral nasal conchae, the third endoturbinate, the vomeronasal organ, the nasal cavernous plexuses, and the lateral nasal gland. The ventral nasal concha is already identified as very small at this level. However, the third endoturbinate reaches its greatest development in this view. The tectorial plate, which occurred in a more rostral view in the cat, also joins more ventrally in the leopard and cheetah to leave the necessary space for the development of the ectoturbinates. From the dorsal to the ventral, the first ectoturbinate develops more laterally, the second more medially, and the third again more laterally. Also notable at this section is the identification of the lateral nasal gland in the four species in the maxillary recess, with an intermediate attenuation in CT and an intermediate signal intensity in MRI.

**Figure 9:**
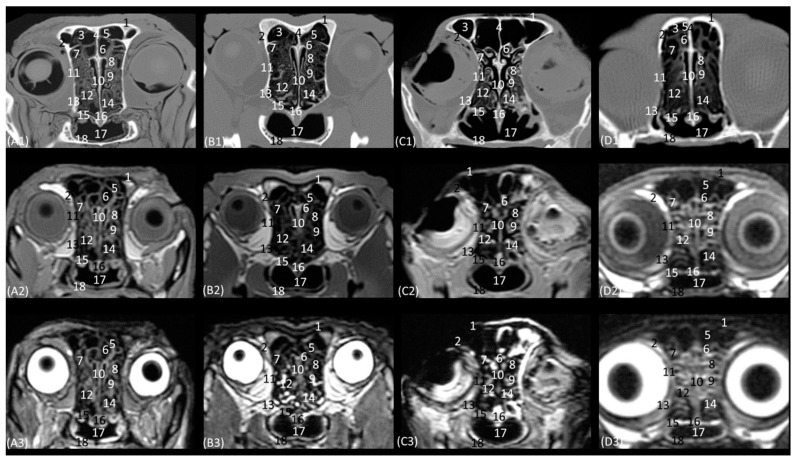
Representative transverse CT (**A1**–**D1**), T1-weighted MR (**A2**–**D2**), and T2-weighted MR (**A3**–**D3**) images at the level of the frontal sinuses and choana. Level section (**A**) VIII. Transverse images are oriented so that the left side of the head is to the right and the dorsal is at the top. All views are rostral. (**A1**–**A3**): Leopard; (**B1**–**B3**): lion; (**C1**–**C3**): cheetah; and (**D1**–**D3**): cat. 1. Frontal bone: squama (external plate); 2. frontal bone: orbital surface; 3. frontal sinus; 4. frontal sinus: septum; 5. 1st ectoturbinate. 6. 2nd ectoturbinate; 7. 3rd ectoturbinate; 8. dorsal nasal concha; 9. middle nasal concha; 10. nasal septum: ethmoid bone (perpendicular plate); 11. ethmoid bone: orbital surface; 12. 3rd endoturbinate; 13. presphenoid bone: wing; 14. 4th endoturbinate; 15. ethmoid bone: basal plate; 16. vomer bone; 17. choana; 18. palatine bone: horizontal and perpendicular plate.

The last transverse plane of the nasal cavity from this CT and MRI study is presented in Figure 9(A1–D3). The bony part of the nasal cavity at this level is represented dorsally by the external plate of the frontal bone; laterally by the orbital surface of the ethmoid bone and the wing of the presphenoid bone; and ventrally by the basal plate of the ethmoid bone, the vomer, and the palatine bone. In addition, ventral to the frontal sinus, the tectorial plate of the ethmoid bone was observed, as was, medially, the bony nasal septum. Ventrally to the nasal cavity, the choanae were observed. The vomer bone ends at this level, and it differs among the species. In the leopard and the lion, the vomer has an oval morphology, while in the cheetah, it is arrow-shaped, and in the domestic cat, it is smaller and rounded. The nasal septum, at this section level, can already be observed as ossified in the four species, hyperattenuated in CT, and in MRI, it has a higher signal intensity than in the more rostral sections. The nasal cavity of this section is made up of three ectoturbinates, the dorsal and middle nasal concha, and the third and fourth endoturbinates. The choanae were observed in all four felids and were delimited dorsally by the basal plate of the ethmoid bone and ventrally by the perpendicular and horizontal plate of the palatine bone. In this last plane, the frontal sinus was seen in the lion, along with the development of the three ectoturbinates, which at this level appeared to be larger in all four species. Furthermore, at this level, we can already identify the fourth endoturbinate in the most ventral part of the nasal cavity.

**Figure 10:**
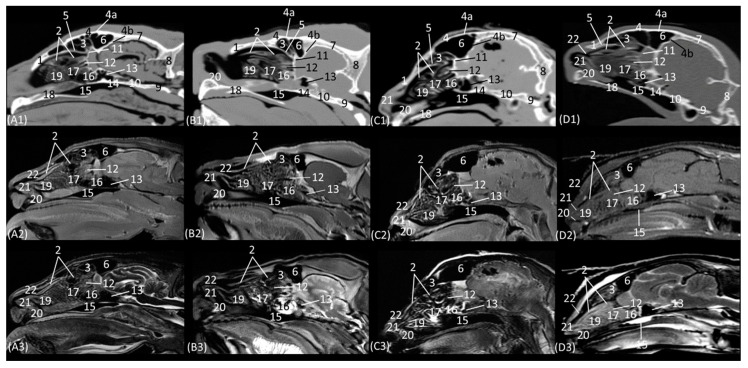
Representative parasagittal multiplanar reconstruction (MPR) CT (**A1**–**D1**), T1-weighted MR (**A2**–**D2**), and T2-weighted MR (**A3**–**D3**) images cutting off the nasal conchae, frontal, and sphenoidal sinuses. Level section (**B**) I. Parasagittal images are oriented so that the rostral part is to the left and the dorsal is at the top. All views are left lateral. (**A1**–**A3**): Leopard; (**B1**–**B3**): lion; (**C1**–**C3**): cheetah; and (**D1**–**D3**): cat. 1. Nasal bone; 2. dorsal nasal concha; 3. 1st, 2nd, and 3rd ectoturbinates; 4. frontal bone: 4a frontal bone: external plate; 4b. frontal bone: internal plate; 5. ethmoid bone: tectorial plate; 6. frontal sinus; 7. parietal bone; 8. occipital bone: squama; 9. occipital bone: basilar part; 10. basisphenoid bone; 11. ethmoid bone: cribiform plate; 12. middle nasal concha; 13. sphenoidal sinus; 14. presphenoid bone; 15. choana; 16. 4th endoturbinate; 17. 3rd endoturbinate; 18. incisive and maxillary bones: palatine process; 19. ventral nasal concha; 20; basal fold; 21. alar fold; 22. straight fold.

Figure 10(A1–D3) represents a sagittal plane of the nasal cavity using CT and MRI. A parasagittal plane was selected to better identify the ethmoidal labyrinth. The entire skull and nasal cavity can be clearly seen. Thus, dorsally and from the rostral to the caudal, we can identify the nasal bone and the external plate of the frontal bone, with the frontal sinus caudally limiting the internal plate of the frontal bone and the tectorial plate of the ethmoid bone. At the caudal limit of the nasal cavity, we can see the cribriform plate of the ethmoid bone and the presphenoid bone with its sinus, and ventral to this, we can see the palatine and the vomer bones and the palatine processes of the maxillary and incisive bones. The rostral limit of the nasal cavity is determined ventrally by the body of the incisive bone, laterally by the nasal processes of the incisive bone, dorsally by the rostral end of the nasal bones, and medially by the cartilage of the nasal septum. The bones appeared to be hyperattenuated on CT, while on MRI, the thin hypointense cortex surrounding the hyperintense medullary bone was visualized throughout all the bones. Within the frontal sinus, the first, second, and third ectoturbinates were observed. In the nasal cavity, we can identify the straight, alar, and basal folds; the dorsal, middle, and ventral nasal conchae; the three ectoturbinates; the third and the fourth endoturbinate; the olfactory bulb; and the nasopharynx. In this view, we can see the beginnings and the course of the dorsal, middle, and ventral nasal conchae, with the latter remaining at more rostral levels and being caudally overlapped by the third endoturbinate, which is greatly expanded in all four species. The nasal conchae were hyperattenuated and slightly hyperintensified at the caudal level due to their bony base. It was also possible to identify more caudally the sphenoid sinus from which the fourth endoturbinate projects. In this plane, the beginning and the entire extension of the nasopharynx were also very well visualized, being observed to be hypoattenuated on CT and hypointense on MRI throughout its entire length. On the other hand, in this section, the straight, alar, and basal folds were clearly identified from dorsal to ventral, observed with attenuation and intermediate intensity.

**Figure 11:**
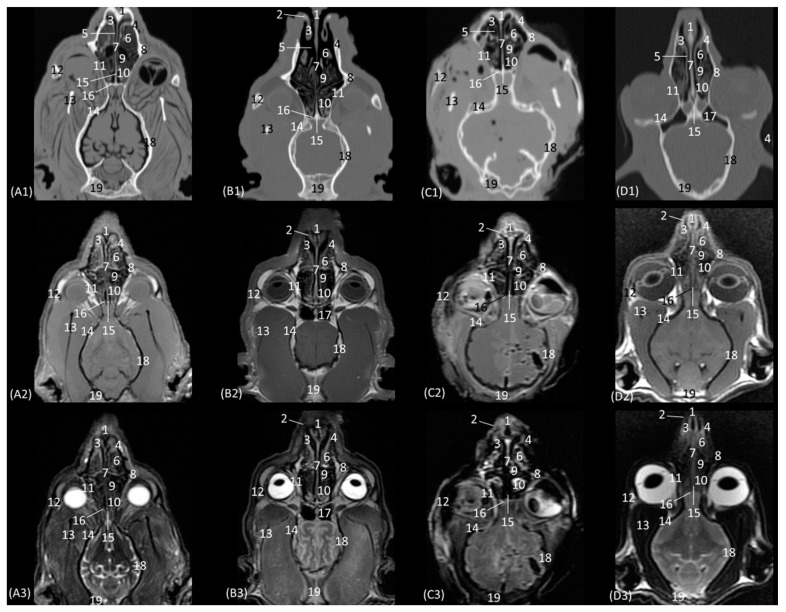
Representative dorsal multiplanar reconstruction (MPR) CT (**A1**–**D1**), T1-weighted MR (**A2**–**D2**), and T2-weighted MR (**A3**–**D3**) images at the level of the ventral nasal concha and perpendicular plate of the ethmoid bone. Level section (**C**) I. Dorsal images are oriented so that the rostral part is to the top. All views are dorsal. (**A1**–**A3**): Leopard; (**B1**–**B3**): lion; (**C1**–**C3**): cheetah; and (**D1**–**D3**): cat. 1. Incisive bone; 2. ventral lateral nasal cartilage; 3. alar fold; 4. maxillary bone; 5. common nasal meatus; 6. ventral nasal concha; 7. nasal septum: cartilage; 8. zygomatic bone: frontal process; 9. 3rd endoturbinate; 10. 4th endoturbinate; 11. frontal bone: orbital surface; 12. zygomatic bone; 13. mandible: ramus; 14. presphenoid bone: wing; 15. ethmoid bone: perpendicular plate; 16. ethmoid bone: cribriform plate; 17. frontal sinus; 18. temporal bone: squamous part; 19. occipital bone: squama.

**Figure 12:**
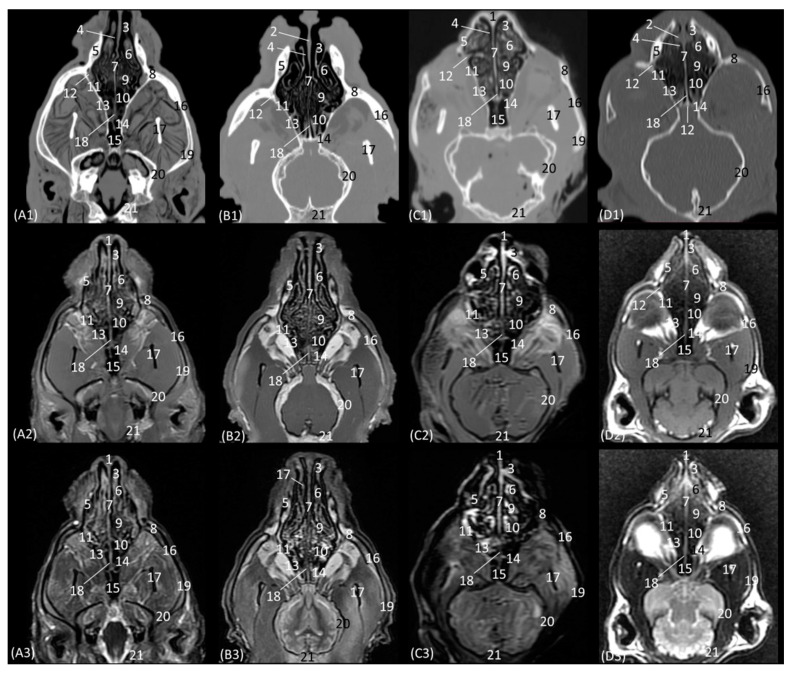
Representative dorsal multiplanar reconstruction (MPR) CT (**A1**–**D1**), T1-weighted MR (**A2**–**D2**), and T2-weighted MR (**A3**–**D3**) images at the level of the 3rd and 4th endoturbinates. Level section (**C**) II. Dorsal images are oriented so that the rostral part is to the top. All views are dorsal. (**A1**–**A3**): Leopard; (**B1**–**B3**): lion; (**C1**–**C3**): cheetah; and (**D1**–**D3**): cat. 1. Incisive bone; 2. nasal cavernous plexuses; 3. alar fold; 4. common nasal meatus; 5. maxillary bone; 6. ventral nasal concha; 7. nasal septum: cartilage; 8. zygomatic bone; 9. 3rd endoturbinate; 10. 4th endoturbinate; 11. lacrimal bone; 12. lateral nasal gland; 13. presphenoid bone: wing; 14. basisphenoid bone: wing; 15. sphenoidal sinus; 16. zygomatic bone: temporal process; 17. mandible: ramus; 18. ethmoid bone: perpendicular plate; 19. temporal bone: zygomatic process; 20. temporal bone: squamous part; 21. occipital bone: squama.

The horizontal or coronal view of the nasal cavity by CT and MRI is represented in Figure 11(A1–D3) and Figure 12(A1–D3). The first dorsal section (I) (Figure 11(A1–D3)) of the nasal cavity was delimited rostrally by the incisive bone, laterally by the maxillary bone, also partially involving the zygomatic and lacrimal bones, as well as the orbital surface of the frontal bone and its zygomatic process, and caudally by the cribriform plate of the ethmoid bone. The nasal septum can be seen as it begins rostrally, appearing as soft tissue due to its cartilaginous nature, and finally ends up ossifying more caudally. In the nasal cavity, we can highlight the alar fold, the ventral nasal concha, and the third endoturbinate. The alar fold can be observed in all four species, as it is part of the ventral nasal concha. The third endoturbinate embraces the ventral nasal concha and is well developed, occupying a large part of the nasal cavity. It was fixed caudally to the cribriform plate of the ethmoid bone. The frontal sinus was only identified in this plane on CT in the domestic cat and on MRI in the lion due to the slight inclination of some sections. The frontal sinus of the lion was divided by the bony septum of the frontal sinus.

Figure 12(A1–D3) represents a ventral horizontal view of the nasal cavity that sections the entire zygomatic arch, the temporal process of the zygomatic bone, as well as the zygomatic process of the temporal bone, although the latter is observed only in the leopard and the cheetah in the study by CT. The maxillary bone was left without rostral continuation in leopard, lion, and cat while in the cheetah, it continued with the incisive bone rostrally. In the nasal cavity, the alar fold, the ventral nasal concha, the third and the fourth endoturbinates, the nasal cavernous plexuses, and the lateral nasal gland were observed. The alar fold was still identified at this level, but in the lion, the basal fold was also visible. The lateral nasal gland was visible in this section in all four species in an area of soft tissue close to the lacrimal bone, which was identified as hypoattenuated on CT and hypointense on MRI with respect to the bone. On the other hand, the sphenoid sinus can be identified in the leopard and the cheetah, although it does not appear in the lion or the cat. Furthermore, at this level and caudal to the fourth endoturbinate, we can identify the sphenoid sinus with a thin septum that was hyperattenuated on CT and hyperintense on MRI in the leopard, cheetah, and cat, but it was not evident in the lion on both CT and MRI, nor in the cat on CT. Finally, unlike the other species, the nasal cavernous plexuses were highly developed in the cat and a little in the lion, and they were located in the most rostral part of the nasal septum.

### 3.2. Rhinoscopy

**Figure 13:**
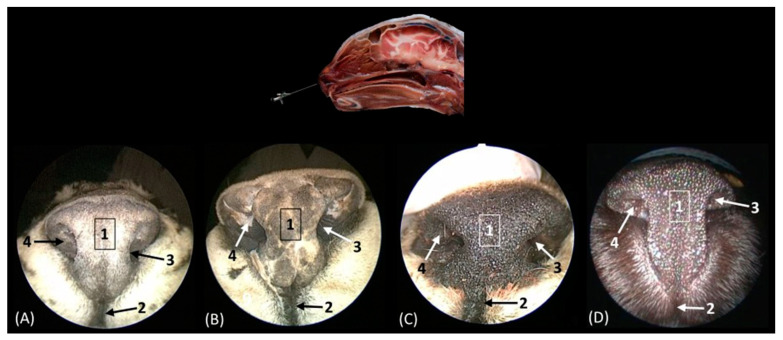
Endoscopic images of the nasal cavity at the level of the nasal orifices. The level of this endoscopic study is shown above in the sagittal anatomical section. Images are observed so that the right side of the head is to the left of the image. (**A**): Leopard; (**B**): lion; (**C**): cheetah; and (**D**): cat. 1. Tip of the nose; 2. subnasal groove; 3. nasal orifice; 4. alar fold.

**Figure 14:**
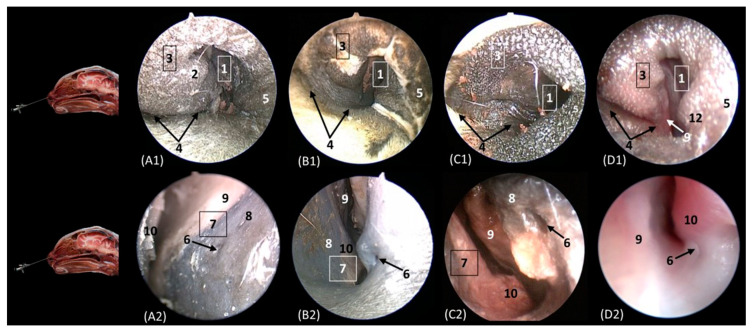
Endoscopic images of the nasal cavity at the level of the nasal vestibule and nasolacrimal duct opening. The levels of this endoscopic study are shown on the left in the sagittal anatomical sections. Images are observed so that the right side of the head is to the left of the image. (**A1**,**A2**): Leopard; (**B1**,**B2**): lion; (**C1**,**C2**): cheetah; and (**D1**,**D2**): cat. 1. Nasal orifice; 2. alar fold; 3. wing of the nose; 4. alar groove; 5. mobile rostral part of nasal septum; 6. nasolacrimal duct opening; 7. mucocutaneous junction of the nose; 8. nasal cavity: nasal vestibule; 9. nasal cavity: respiratory part; 10. basal fold.

**Figure 15:**
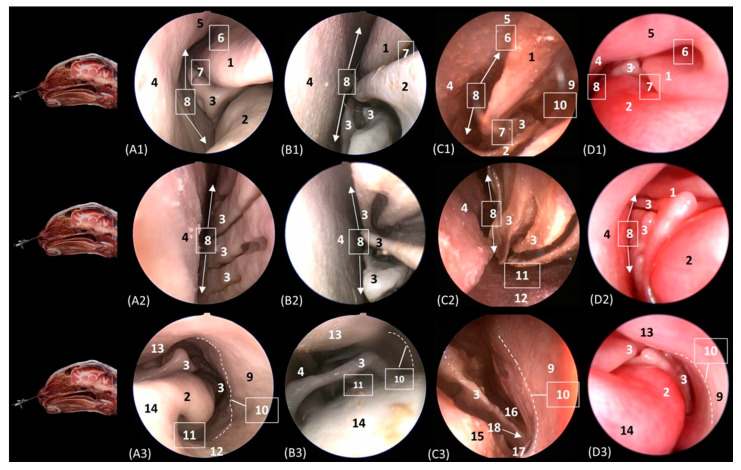
Endoscopic images of the nasal cavity at the level of the dorsal and ventral nasal conchae. The levels of this endoscopic study are shown on the left in the sagittal anatomical sections. Images are observed so that the right side of the head is to the left of the image. (**A1**–**A3**): Leopard; (**B1**–**B3**): lion; (**C1**–**C3**): cheetah; and (**D1**–**D3**): cat. 1. Dorsal nasal concha; 2. ventral nasal concha; 3. 3th endoturbinate; 4. nasal septum; 5. nasal cavity: roof; 6. dorsal nasal meatus: 7. middle nasal meatus; 8. common nasal meatus; 9. nasal cavity: lateral wall; 10. maxillary recess; 11. ventral nasal meatus; 12. nasal cavity: floor; 13. straight fold; 14. alar fold; 15. basal fold; 16. dorsal part of maxillary recess; 17. ventral part of maxillary recess; 18. beginning of ventral nasal concha in the conchal crest of the maxillary bone.

**Figure 16:**
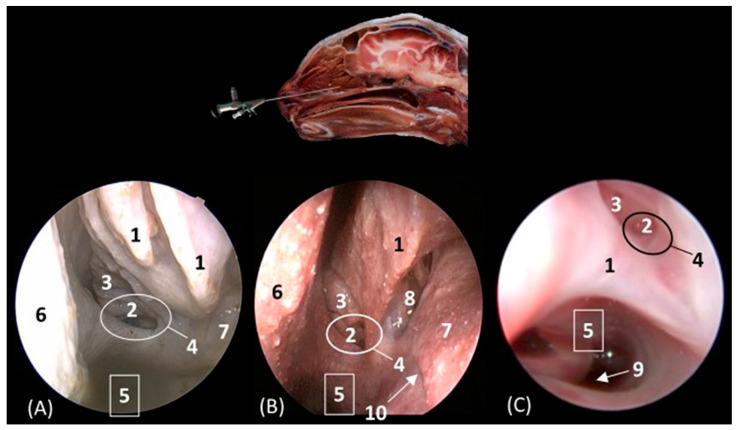
Endoscopic images of the nasal cavity at the level of the ethmoidal labyrinth. The level of this endoscopic study is shown above in the sagittal anatomical section. Images are observed so that the right side of the head is to the left of the image. (**A**): Lion; (**B**): cheetah; and (**C**): cat. 1. Ventral nasal concha; 2. 4th endoturbinate; 3. 3rd endoturbinate; 4. opening of the sphenoidal sinus; 5. ventral nasal meatus; 6. nasal septum; 7. nasal cavity: lateral wall; 8. lateral nasal gland; 9. choana; 10. pharyngeal orifice of the auditory tube.

**Figure 17:**
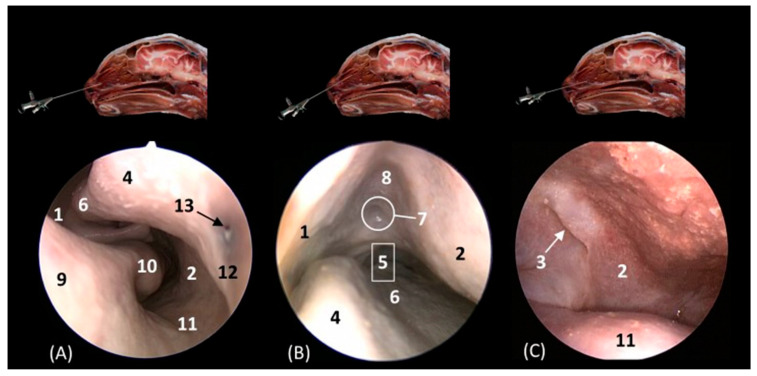
Endoscopic images of the nasal cavity showing the aperture of the lateral nasal gland duct, frontal opening, and pharyngeal orifice of the auditory tube (observed only in big cats). The levels of this endoscopic study are shown above in the sagittal anatomical sections. Images are observed so that the right side of the head is to the left of the image. (**A**): Leopard; (**B**): lion; and (**C**): cheetah. 1. Nasal septum; 2. nasal cavity: lateral wall; 3. pharyngeal orifice of the auditory tube; 4. straight fold; 5. dorsal nasal meatus; 6. dorsal nasal concha; 7. frontal opening; 8. nasal cavity: roof; 9. alar fold; 10. ventral nasal concha; 11. nasal cavity: floor; 12. oblique fold; 13. aperture of the lateral nasal gland duct.

**Figure 18:**
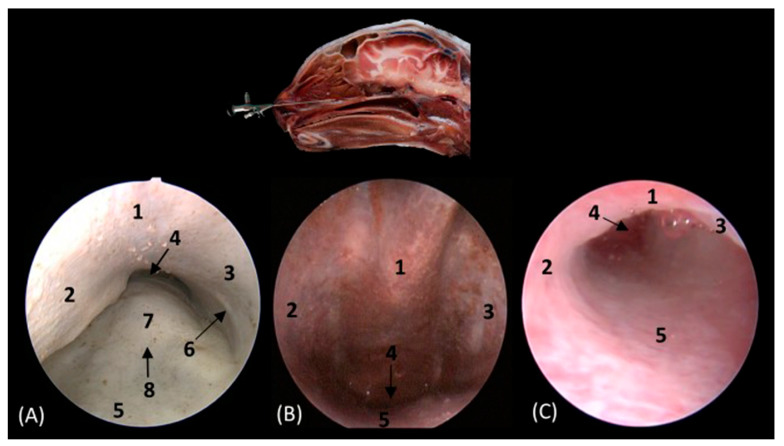
Endoscopic images at the level of the choana and nasopharynx. The level of this endoscopic study is shown above in the sagittal anatomical section. Images are observed so that the right side of the head is to the left of the image. (**A**): Lion; (**B**): cheetah; and (**C**): cat. 1. Presphenoid bone; 2. nasal septum; 3. nasal cavity: lateral wall; 4. choana; 5. nasal cavity: floor; 6. pharyngeal orifice of the auditory tube; 7. soft palate; 8. palatine aponeurosis.

The rhinoscopic study of the nasal cavity is seen in Figure 13, Figure 14, Figure 15, Figure 16, Figure 17 and Figure 18. Figure 13 shows the nasal plane (the external nose) of the four felids. The vertex of the nose of the leopard and lion resemble each other in morphology, exhibiting an inverted triangle shape. However, in the cheetah, this shape is much more rectangular, and in cats, it is more elongated. The subnasal groove can be observed in the four species ventrally to the external nose. The alar fold was observed in all four species, and in the leopard and the lion, it extends lateromedially to the middle of the external nose, while in the cheetah, it runs almost the entire extent of the external nose; in cats, however, it ends more dorsally. The development of this fold also surrounds and shapes the nostrils. In the cheetah, this nasal orifice is larger than in the rest of the felids that we studied due to the conformation of its alar fold.

Figure 14 shows the nasal vestibule, the most rostral part of the nasal cavity. In this and subsequent planes, the rhinoscope was introduced into the left nostril. In Figure 14 (A1–D1), the path of the alar fold was observed in all four species, forming the wing of the nose and ventrally forming the alar groove, which develops deeper in the leopard and the lion. As previously mentioned, the width of the nostril depends on the morphology of the alar fold, exhibiting a greater width in the cheetah. Figure 14(A2–D2) show the nasal vestibule in more detail. We can see the mucosa of the nasal vestibule and the mucosa of the nasal cavity, as well as the mucocutaneous junction between them. This transition is much more gradual in domestic cats than in big cats. The orifice of the nasolacrimal duct was identified close to this junction, on the floor of the vestibule. It was observed that this orifice is larger in cheetah than in the other species. Lateral to this opening, the end of the basal fold is seen.

Figure 15 delves into the respiratory portion of the nasal cavity. Three views were observed in the four species, from dorsal to ventral. In the most dorsal view (Figure 15(A1–D1)), the development of the dorsal nasal concha can be seen, starting from the lateral side of the nasal cavity. Ventral to it, the ventral nasal concha was observed, but between the two conchae, the third endoturbinate was also seen. The meatuses can also be observed between the conchae. The dorsal nasal meatus was visualized between the roof of the nasal cavity and the dorsal nasal concha. The middle nasal meatus was located between the dorsal nasal concha and the ventral nasal concha, while the ventral nasal meatus appeared between the ventral nasal concha and the floor of the nasal cavity. Finally, the common nasal meatus was identified between the medial surface of the nasal conchae and the nasal septum. These meatuses were described as narrower in the domestic cat than in the rest of the studied felids. Furthermore, it is worth highlighting how in the cheetah and the lion, the third endoturbinate presents a more sinuous, narrow, and flattened morphology than in the other species. In a more ventral view, the development of the maxillary recess was observed, located laterally to the ventral nasal concha (Figure 15(A1–D1)).

Figure 16A–C represent a more caudal view at the level of the ethmoidal labyrinth. Only views of the lion, cheetah, and cat are included, as due to the rigidity of the fixed structures of the nasal cavity of the leopard, access by rhinoscopy for a more caudal study of the nasal cavity is presented. In this section, the third and fourth endoturbinates were identified, as well as the opening of the sphenoid sinus, which is close to the floor of the nasal cavity. In the cat, the nasopharyngeal or choanal opening can also be identified ventrally, and in the cheetah, the pharyngeal orifice of the auditory tube can be identified on the lateral wall of the nasal cavity, ventral to the third and fourth endoturbinates.

The pharyngeal orifice of the auditory tube can be identified in more detail in Figure 17C, located on the lateral wall. The leopard is the only species where the orifice of the lateral nasal gland was identified, being located close to the straight fold and, more specifically, in the oblique fold (Figure 17A).

Below, Figure 18A–C detail the nasopharyngeal or choanal opening in the lion, cheetah, and cat on the floor of the nasal cavity, showing the soft palate at this level. In this view, we can also see the pharyngeal opening of the auditory tube on the lateral wall of the nasal cavity.

## 4. Discussion

### 4.1. Anatomical, Comparative, and Functional Considerations

#### 4.1.1. External Nose

In the nasal plane, the width of the nostril depends on the morphology of the alar fold. Thus, in the leopard and the cheetah, a smaller extension of this fold was observed in both CT and MRI, which means that on observation of the nasal plane of these felids by rhinoscopy, the lion and cat presented a narrower orifice than the leopard and cheetah did. The rostral nasal plane of felids was not described in any study using diagnostic imaging techniques and rhinoscopy published to date. For its study and for that of its cartilaginous structure, we have consulted anatomical texts [6,38,39,41] and used rhinoscopic vision.

#### 4.1.2. Nasal Vestibule

In this plane, it worth nothing that, due to the more caudal projection of the alar fold in lions and cats, the incisive bone and teeth can already be visualized, while at the same level of the nasal section as in the leopard and cheetah, we can only see the alar fold. Parallel folds [6,38,39] are structures that are only visualized in a very rostral plane, and in our study, it was only possible to identify them with CT and MRI in the leopard and the cheetah.

The straight, alar, and basal nasal folds can be identified in the four species in our study, as Conchou et al. [9] do in the domestic cat. However, Losonsky et al. [10] only identified the alar fold in the cat. In addition, the same authors call the basal fold the “caudal portion of the alar fold”.

#### 4.1.3. Nasal Cavity

The nasal cavity of the domestic cat is similar to that of nondomestic cats; however, certain differences are worth highlighting. Unlike the big cats studied here, the domestic cat has two “conchal crests of the maxillary bone” for anchoring the ventral nasal concha to the internal wall of the maxillary bone. In the leopard, the lion, and the cheetah, we observed how the ventral nasal concha is attached to the lateral wall of the nasal cavity by only one crest. For its part, the dorsal nasal concha is attached to the lateral wall of the nasal cavity by the ethmoidal crest of the nasal bone. These findings were not described in any study to date.

In the lion, we observed that the internasal symphysis (medial union of the nasal bone) appeared to be much more caudal than in the rest of the feline species (Figure 4 and Figure 5), so its mucosa and cartilaginous structure at this level (the dorsal lateral cartilage) must be quite rigid and resistant in the most rostral planes.

With respect to the nasal cavernous plexuses, it should be noted that they were neither described by Losonsky et al. [10], nor by Conchou et al. [9]. Schaller [38] described them in bovids as a set of dense venous networks in the submucosa, adjacent to both the nasal septum, as well as the lateral floor of the nasal cavity. Sandoval [39] also identified them without indicating a specific species and described their drainage into the sphenopalatine vein. We can identify these plexuses in our study next and laterally to the nasal septum. In the species of big cats that we studied, the plexuses were much more developed in the leopard than in the lion or the cheetah. We can relate this to the leopard’s need to heat inhaled air, as they can withstand temperatures ranging between −30 and +70 °C [16]. In the lion and the cheetah, this is not necessary, as their habitats are not so extreme, and consequently, their plexuses are less developed [15,17].

Regarding the nasal conchae and the ecto- and endoturbinates, neither Losonsky et al. [10], nor Conchou et al. [9] described the middle nasal concha and the third and fourth endoturbinates. They mentioned ethmoturbinates in passing. Additionally, Losonsky et al. [10] described the dorsal nasal concha, where the second ectoturbinate was located in the frontal sinus. In our study, using both CT/MRI and rhinoscopy, in the lion and the cheetah, we observed that the nasal conchae and endoturbinates were more tightly coiled than in the other studied felids, thus leaving wider meatuses. This may also be related to the speed that these two felids can reach when running, since the cheetah is the fastest feline species [17].

Furthermore, another peculiarity that we observed in the lion throughout this study was the great thickness of the bones of the roof of its nasal cavity, mainly the frontal and nasal bones, as well as the great thickness of the muscles of its head. This may be related to the degree of resistance to blows and impact in the head region by large herbivores (buffalos, giraffes, zebras) when they defend themselves from attacks with their horns and by kicking for territory and food. It is also important to highlight the effect that captivity has on the lower development of the trabecular bone in felids [42].

The maxillary recess was only identified using imaging techniques with CT and MRI by De Rycke et al. [11] and with CT by Weeden et al. [1], which are both studies on the dog. In our study, the recess was visible in CT, MRI, and rhinocopy, limited medially by the ethmoidal labyrinth, laterally by the lacrimal and maxillary bone, and lateroventrally by the palatine bone.

The frontal sinus in cats, according to Barone [6] and Conchou et al. [9], has two parts (a rostral and a caudal part). However, Schaller, Sandoval, Vázquez et al. [38,39,43] described a single frontal sinus in felids. Thus, in our study, it can be seen how the tectorial lamina of the ethmoid bone covers and dorsally fixes the ectoturbinates within the frontal sinus, and it appeared to divide the frontal sinus, but in fact, it was a single sinus. In the domestic cat, the opening of the frontal sinus was identified using CT, which joined the frontal sinus with the nasal cavity and indirectly with the maxillary recess. Furthermore, using the rhinoscopic technique, in the lion, we also identified the opening of the sinus in the root of the nasal cavity. In addition, it should be noted that this opening only opens directly to the caudal maxillary sinus in equids, receiving the name of the frontomaxillary opening [38,39,43].

It should be noted that the lateral nasal gland had not yet been identified using diagnostic imaging techniques. In our study, with the help of Barone [6] and Sandoval [39], we were able to locate it using CT, MRI, and rhinoscopy. Rhinoscopy has allowed us to observe its location in the lion, the cheetah, and the cat, since it is camouflaged between the third endoturbinate and the maxillary recess. In addition, this also allowed us to locate the orifice of this gland, at the caudal limit of the nasal vestibule in the leopard.

#### 4.1.4. Nasopharynx

Regarding the nasopharynx, the nasal cavity communicates with the nasal portion of the pharynx through the choanae or nasopharyngeal openings [43]. These openings have been identified in the four species of felids by CT and MRI; however, using rhinoscopy, we were only able to visualize them in the lion, cheetah, and cat due to the rigidity of the anatomical structures caused by fixation, preventing the passage of the rhinoscope to the most caudal regions. Close to the choanae, we identified on the lateral wall of the nasopharynx the pharyngeal orifice of the auditory tube in both the lion and the cheetah, which has not been identified to date in any rhinoscopic study. The nasopharynx has a more flattened lumen in the domestic cat than in the rest of the big cats that we studied, a fact that is very well illustrated in both the transverse and sagittal views of CT and MRI.

### 4.2. Clinical and Pathological Considerations

Neoplasms, inflammatory and non-inflammatory diseases, trauma, dental diseases, and nasopharyngeal diseases can cause clinical signs that are localized to the feline nasal cavity and paranasal sinuses. A detailed knowledge of the normal anatomy that is specific to the feline species facilitates the diagnosis and characterization of the lesions and abnormalities that can appear in the nasal cavity and paranasal sinuses [10]. Furthermore, most tumors of the nasal cavity are locally invasive and malignant in dogs and cats; however, some of them can be resected by surgery, and it is important to know the specific anatomy of the nasal cavity [1]. Weeden et al. [1] present in their study an anatomical study of the dog’s nasal cavity using CT with a surgical approach. Regarding dogs, it is also worth highlighting the study carried out by De Rycke et al. [11], who developed a study of the nasal cavity of this species using CT, MRI, and anatomical sections.

In the domestic cat, there are various studies of the nasal cavity using diagnostic imaging techniques. Specifically, Conchou et al. [9] present an atlas of the nasal cavity and paranasal sinuses of the cat using MRI. However, they presented only transverse and horizontal views of the nasal cavity. Furthermore, in our study, we could visualize structures that Conchou et al. [9] did not identify (among them, the third and fourth endoturbinate, the maxillary recess, and the lateral nasal gland). Losonsky et al. [10] carried out a similar study, but using CT, highlighting the scarcity of sections that have been studied and the lack of definition of the tomographic images to identify the structures, which required them to present a schematic drawing to identify many of the anatomical features.

In relation to the study of the nasal cavity by rhinoscopy, we can highlight other studies which describe the procedure to carry out a rhinoscopic study in dogs and cats [3,14]. McCarthy et al. [18] also described the rhinoscopic procedure and also added a small anatomical description of the nasal cavity, but only attaching a few anatomical images, some of which were of poor quality.

As far as big cats are concerned, to date, there are no studies of the anatomy of the nasal cavity using imaging techniques. Snow et al. [30] presented an MRI study of the tiger head but did not delve into the nasal cavity. Jáber et al. [32] also performed a study of the Bengal tiger´s brain using MRI, but it does not describe the nasal cavity either. There are very few studies published to date of clinical cases that affect the nasal cavity in big cats. We can cite some relevant studies on clinical cases, such as the one carried out by Van der Weyden et al. [35], which proposed a clinical case of a meningioma of the nasal cavity of a Siberian tiger without the prior use of imaging techniques, which was diagnosed post mortem in the necropsy; or the work of Noguchi et al. [44], which described a clinical case of a cheetah involving excision of a nasopharyngeal nodule, diagnosed with CT and removed by rhinoscopy. Similarly, Ready et al. [36] described clinical cases of melanocytic neoplasms in tigers, African lions, and snow leopards that, among other locations, affect the nasal plane, and their study used radiographic and tomographic studies.

In this study, the advantages and disadvantages of studying the nasal cavity using CT, MRI, and rhinoscopy can be observed. To date, there are no such exhaustive studies of the nasal cavity that encompass both imaging techniques and rhinoscopy. CT allowed the bony limits and extensions of the nasal cavity to be identified much better than with MRI, since, in their entirety, they are completely hyperattenuated. With MRI, the bone was observed with different signal intensities depending on whether it was cortical or medullary, which in many cases makes it difficult to visualize the bone limits. The cortical layer, due to its low concentration of hydrogen atoms, was observed to be hypointense, while the medullary, as it contains bone marrow, which is rich in fat and therefore has a greater amount of hydrogen atoms, was observed to be hyperintense. In contrast, the MRI study provided better detail of the soft tissue, which allowed for clearer observation of the entire development of the turbinates, vomeronasal organ, lateral nasal gland, and nasal cavernous plexuses. Furthermore, with T2-weighted MRI, fluids and edema can be visualized as hyperintense, which can help in the interpretation of pathological conditions [9]. The rhinoscopic study, for its part, allowed for a direct visualization of the nasal cavity; however, it has the limitation that it cannot access all structures due to the restriction that rhinoscope access can pose in many areas. In our study, due to the rigidity after preservation with fixative solution of the leopard’s head, it was impossible to access the more caudal levels. In this context, it is also worth highlighting the different colors of the mucosa of the nasal cavity of the domestic cat compared to the big cats, which was shown to have a much more vivid and pink color in the cat, “as it is a living animal”. This is due to the effect of the fixative solution on the mucosa, since the rhinoscopic study of big cats was performed, unlike in the domestic cat, on fixed specimens.

The information provided in this article was intended to be key to the identification of pathological alterations in the nasal cavity. Diseases such as inflammatory rhinitis can cause hyperplasia of the nasal mucosa, as well as an increase in secretions [2]. Among the origins of rhinitis, we can highlight fungal conditions, for which the definitive diagnosis is taking biopsies through rhinoscopy [21]. Nasopharyngeal masses are also common in felids, usually of tumor origin, and their location and limits can be observed with considerable precision by both CT and MRI. Foreign bodies can also be identified in the nasal cavity, which are mainly diagnosed by CT and rhinoscopy [26]. Additionally, abscesses in the roots of the maxillary canine teeth can cause nasal discharge if the abscess extends to the nasal cavity [2]. The maxillary sinus is frequently affected when an abscess is generated due to infection of the caudal maxillary premolar teeth [1]. In this context, it is also worth noting that after having visualized the connection of the maxillary recess with the frontal sinus in the cat through the opening of the frontal sinus in our study, a condition at the level of these teeth could even develop into sinusitis or infection in this sinus.

All these pathologies that affect the nasal cavity, if they become chronic, can lead to the destruction of structures of the nasal cavity such as the turbinates [2]. Therefore, prior knowledge of nasal anatomy is necessary for the diagnosis of any nasal condition [9,10,14].

## 5. Conclusions

The combination of CT, MRI, and rhinoscopy techniques for examining the nasal cavity has allowed us to assess the benefits that each technique has for the study of this cavity.

CT is a technique of great value for the study of the bony nasal cavity, since the bones are visualized in their entire thickness in a hyperattenuated manner, which facilitates the identification of their limits. However, MRI presents certain difficulties for this, since it shows the bones with a hypointense cortical and a hyperintense medullary, which makes visualization difficult in many cases. In addition, MRI is very useful in visualizing soft tissue, which includes the mucosa of the turbinates or conchae, as well as structures such as the vomeronasal organ or the lateral nasal gland.

Rhinoscopy is a key technique in the direct visualization of the mucosa of the nasal vestibule and nasal cavity and allows for the differentiation of some structures such as the orifice of the nasolacrimal duct, the pharyngeal orifice of the auditory tube, or the orifice of the lateral nasal gland, which cannot be identified with any other imaging technique used.

In the external nose, the opening of the nostrils depends on the route and morphology of the alar fold, a fact that causes the lion and cat to have narrower nostrils.

At the level of the nasal cavity, it has been observed that the ventral nasal concha is attached to the lateral wall of the nasal cavity by a single conchal crest of the maxillary bone in big cats, while in domestic cats, it has two conchal crests. On the other hand, the attachment of the dorsal nasal concha to the lateral wall of the nasal cavity is through the ethmoidal crest of the nasal bone. In lions, we could highlight that the medial union of the nasal bone or internasal symphysis is much more caudal than in the rest of the feline species that we studied. Furthermore, the bones of the nasal cavity of the lion, as well as the muscles of this area, are much thicker in this species than in the others.

The nasal cavernous plexuses are more developed in leopards than in the rest of the species.

The nasal conchae, as well as the endoturbinates, present a narrower and flatter morphology in the lion and the cheetah, which means that the spaces between them, or the nasal meatuses, have a greater width.

With respect to the frontal sinus, in the domestic cat, it has been observed that the opening of the frontal sinus connects the frontal sinus with the nasal cavity and, indirectly, with the maxillary recess. This particularity has not been found in big cats.

The nasopharynx has a much narrower lumen in the domestic cat than in the big cats.

Finally, we can conclude by emphasizing that adequate knowledge of the specific anatomy of the nasal cavity of these species through imaging techniques allows us to more easily detect and diagnose alterations or pathologies that may affect this level.

## Data Availability

The information can be requested from dlcasas@gmail.com, almoradi@mivet.com, and ricardo.n.l@um.es.

## Supplementary Materials

The following supporting information can be downloaded at https://www.mdpi.com/article/10.3390/ani14081172/s1: Table S1: MRI parameters used in this study; Table S2: Tissue CT density and signal intensity characteristics for this CT and MRI study.
