# Supplementary material for: An Anatomical Study Using Computed Tomography, Magnetic Resonance Imaging, and Rhinoscopy of the Nasal Cavity of Domestic Cat (Felis silvestris catus L.) and Big Cats: Lion (Panthera leo leo L.), Leopard (Panthera pardus kotiya L.), and Cheetah (Acinonyx jubatus jubatus S.)
Source: Animals (Basel). 2024 Apr 13;14(8):1172. doi: 10.3390/ani14081172 (PMC11047709; doi:10.3390/ani14081172)
Supplement: Supplementary file 1 [file animals-14-01172-s001.zip › TABLE S1.pdf]

**Table S1.** MRI parameters used in this study.

| Study code | Weighted | Pulse sequence | Dimensional plane | Acquisition | TE | TR | TI | NEX | Slice thickness | Interslice gap | Field of view | Matrix dimensions |
|---|---|---|---|---|---|---|---|---|---|---|---|---|
| LEOPARD | T1 | SE | AXIAL | 2D | 14 | 340 | 0 | 1 | 3 | 3.5 | 100 | 0\320\224\0 |
| LEOPARD | T2 | FSE | AXIAL | 2D | 88.1 | 3700 | 0 | 1 | 3 | 3.5 | 75 | 0\320\256\0 |
| LEOPARD | T1 | SE | SAGITTAL | 2D | 14 | 440 | 0 | 1 | 4 | 4.1 | 75 | 0\320\224\0 |
| LEOPARD | T2 | FRFSE | SAGITTAL | 2D | 106.3 | 6040 | 0 | 1 | 4 | 4.1 | 100 | 384\0\0\224 |
| LEOPARD | T1 | SE | CORONAL | 2D | 14 | 460 | 0 | 1 | 3.5 | 3.8 | 75 | 0\320\256\0 |
| LEOPARD | T2 | FRFSE | CORONAL | 2D | 88.5 | 3820 | 0 | 1 | 3.5 | 3.8 | 75 | 0\320\224\0 |
| LION | T1 | SE | AXIAL | 2D | 14 | 300 | 0 | 1 | 3 | 3.5 | 75 | 0\320\224\0 |
| LION | T2 | FSE | AXIAL | 2D | 87 | 6000 | 0 | 1 | 3 | 3.5 | 75 | 0\320\256\0 |
| LION | T1 | SE | SAGITTAL | 2D | 14 | 240 | 0 | 1 | 4 | 4.1 | 75 | 0\320\224\0 |
| LION | T2 | FRFSE | SAGITTAL | 2D | 114.3 | 4820 | 0 | 1 | 4 | 4.1 | 100 | 514\0\0\256 |
| LION | T1 | SE | CORONAL | 2D | 14 | 300 | 0 | 1 | 3.5 | 3.8 | 75 | 0\320\256\0 |
| LION | T2 | FRFSE | CORONAL | 2D | 87 | 4700 | 0 | 1 | 3.5 | 3.8 | 75 | 0\320\224\0 |
| CHEETAH | T1 | SE | AXIAL | 2D | 14 | 480 | 0 | 1 | 3 | 3.1 | 75 | 0\320\224\0 |
| CHEETAH | T2 | FRFSE | AXIAL | 2D | 105.2 | 4460 | 0 | 1 | 4 | 4.1 | 75 | 384\0\0\224 |
| CHEETAH | T1 | SE | SAGITTAL | 2D | 14 | 320 | 0 | 1 | 4 | 4.1 | 100 | 0\320\224\0 |

| | | | | | | | | | | | | |
|---|---|---|---|---|---|---|---|---|---|---|---|---|
| CHEETAH | T2 | FRFSE | SAGITTAL | 2D | 105.2 | 4460 | 0 | 1 | 4 | 4.1 | 100 | 384\0\0\224 |
| CHEETAH | T1 | SE | CORONAL | 2D | 14 | 400 | 0 | 1 | 3.5 | 3.8 | 75 | 0\320\256\0 |
| CHEETAH | T2 | FRFSE | CORONAL | 2D | 92.8 | 3800 | 0 | 1 | 3.5 | 3.8 | 75 | 0\320\224\0 |
| CAT | T1 | SE | AXIAL | 2D | 14 | 480 | 0 | 1 | 3 | 3.1 | 75 | 0\320\224\0 |
| CAT | T2 | FSE | AXIAL | 2D | 92.3 | 3000 | 0 | 1 | 3 | 3.1 | 75 | 0\320\256\0 |
| CAT | T1 | SE | SAGITTAL | 2D | 14 | 360 | 0 | 1 | 2 | 2.1 | 75 | 0\320\224\0 |
| CAT | T2 | FRFSE | SAGITTAL | 2D | 109.1 | 2200 | 0 | 1 | 2 | 2.1 | 100 | 384/0/0/224 |
| CAT | T1 | SE | CORONAL | 2D | 14 | 280 | 0 | 1 | 3 | 3.1 | 75 | 0\320\256\0 |
| CAT | T2 | FRFSE | CORONAL | 2D | 94.4 | 4840 | 0 | 1 | 3 | 3.1 | 75 | 0\320\224\0 |

*se:* Spin echo sequence; *FrFse:* Fast Recovery Fast Spin Echo sequence.
