# Supplementary material for: An Anatomical Study Using Computed Tomography, Magnetic Resonance Imaging, and Rhinoscopy of the Nasal Cavity of Domestic Cat (Felis silvestris catus L.) and Big Cats: Lion (Panthera leo leo L.), Leopard (Panthera pardus kotiya L.), and Cheetah (Acinonyx jubatus jubatus S.)
Source: Animals (Basel). 2024 Apr 13;14(8):1172. doi: 10.3390/ani14081172 (PMC11047709; doi:10.3390/ani14081172)
Supplement: Supplementary file 1 [file animals-14-01172-s001.zip › TABLE S2.pdf]

**Table S2.** Tissue CT density and signal intensity characteristics for this CT and MRI study.

| TISSUES | CT | Spin-echo T1-weighted | Spin-echo T2-weighted |
|---|---|---|---|
| • **BONES:** Frontal; Nasal; Incisive; Maxillary; Lacrimal, Zigomatic; Vomer and Etmoidal | | | |
| • **Cortical** | Hyperattenuated | Very low | Very low |
| • **Bone marrow** | Intermediate | High | Intermediate |
| • **EXTERNAL NOSE:**<br><br>- Nostril<br>- Wing of the nose<br>- Subnasal groove<br>- Dorsal lateral nasal cartilage<br>- Ventral lateral nasal cartilage<br>- Lateral accessory nasal cartilage | Intermediate | Intermediate | Intermediate |
| • **NASAL CAVITY:**<br><br>- Alar groove<br>- Choanae<br>- Nasal septum cartilage<br>- Vomeronasal organ<br>- Nasal vestibule<br>- Straight fold<br>- Alar fold<br>- Basal fold<br>- Parallel folds<br>- Dorsal nasal concha<br>- Middle nasal concha<br>- Ethmoidal conchae<br>- Ventral nasal concha<br>- Lateral nasal gland<br>- Nasal cavernous plexuses | Intermediate | Intermediate | Intermediate |
| - Dorsal nasal meatus<br>- Middle nasal meatus<br>- Ventral nasal meatus<br>- Common nasal meatus<br>- Ethmoidal meatuses | Hypoattenuated | Very low | Very low |
| • **PARANASAL SINUSES:**<br><br>- Maxillary recess<br>- Frontal sinuses<br>- Frontal sinuses septum<br>- Sphenoidal sinus | Hypoattenuated | Very low | Very low |
